# Factors associated with the presence of anti-*Leptospira* spp. antibodies in persons experiencing homelessness in Brazil

**DOI:** 10.3389/fpubh.2025.1596684

**Published:** 2025-07-11

**Authors:** Haroldo Greca Junior, Cassiana Dahlke Machado, Louise Bach Kmetiuk, Danilo Alves de França, Nassarah Jabur Lot Rodrigues, Anahi Chechia do Couto, Helio Langoni, Gustavo Monti, Alexander Welker Biondo

**Affiliations:** ^1^Department of Veterinary Medicine, Federal University of Paraná, Curitiba, Brazil; ^2^City Secretary of Health, Curitiba, Brazil; ^3^Department of Veterinary Hygiene and Public Health, São Paulo State University, Botucatu, Brazil; ^4^Quantitative Veterinary Epidemiology Group, Wageningen University and Research, Wageningen, Netherlands

**Keywords:** leptospirosis, seroprevalence, homeless populations, risk factors, microscopic agglutination test

## Abstract

**Introduction:**

Homelessness has been considered one of the most vulnerable situations worldwide, alongside people private of liberty (incarceration) and country displacement (refugees). Structural inequality and exposure to diseases such as leptospirosis may be aggravated by individual issues including drug addiction, mental disorders and improper healthcare.

**Methods:**

The present study has accessed persons experiencing homelessness to *Leptospira* spp. exposure by microscopic agglutination test (MAT) for 30 serovars. This study was conducted in São Paulo city in southeastern Brazil and São José dos Pinhais city, belonging the eighth biggest metropolitan area of Brazil in Southern region.

**Results:**

In total, 21/243 (8.6%; 95% CI = 5.6–13.1%) persons experiencing homelessness were seropositive in the MAT. Location, condom use, and flea infestations were identified as statistically significant associated risk factors for exposure.

**Discussion:**

The presence of multiple *Leptospira* spp. serovars may indicate bacterial diversity, even in urban settings. The results herein found for persons experiencing homelessness were not a surprise, as Brazil has been historically recognized as an endemic country for leptospirosis, with 3,810 leptospirosis cases on average per year and the majority living in densely populated urban areas. Multidisciplinary efforts and integrated policies may be crucial to mitigate leptospirosis and other infectious diseases in persons experiencing homelessness, as social neglection may impact on their fundamental rights to dignity and access to personal health.

## 1 Introduction

Leptospirosis, caused by the pathogenic *Leptospira* spp. (1), is one of the most neglected tropical diseases of public health importance worldwide (2). *Leptospira* can survive in the renal tubules of reservoirs, such as domestic and wild animals, and are intermittently shed through urine (3). Contaminated urine, water, and soil are reported sources of human infection through direct contact with the mucous membranes and conjunctivae, particularly during flooding events in urban settings (4). Human leptospirosis may vary in characteristics, with up to 90% of affected individuals having asymptomatic, subclinical, or mild illnesses and the remaining 10% of infected individuals experiencing sudden onset of fever, headache, muscle aches, gastrointestinal symptoms, and multiorgan involvement and failure, resulting in high mortality (4).

Leptospirosis is an occupational disease that directly affects workers through contact with infected animal reservoirs, such as dairy farmers and slaughterhouse workers, and indirectly through contact with individuals exposed to accumulated contaminated water, such as recyclers, bricklayers, sewer cleaners, and other sanitary workers (5). Epidemiologically, a high incidence of human leptospirosis has been reported, mostly through contaminated water or soil, with no conclusive evidence of direct animal contact as an important transmission pathway (6). Socio-environmental conditions, such as inadequate infrastructure and sanitation, contaminated water and soil, waste accumulation, and rodent proliferation, have favored the spread of leptospirosis among vulnerable populations living in the slums of Brazil and other tropical countries (7, 8).

Homelessness may be defined as persons experiencing a lack of permanent housing, mostly living on the streets, and relying on public and private shelters for overnight sleep (9). These groups are highly vulnerable to drug addiction, marginalization, interpersonal violence, and social exclusion. Additionally, worldwide, they are reported to have a high prevalence of infectious and mental diseases (10, 11). The current homeless population in Brazil, estimated to be around 327,000, mostly live in poor conditions and highly densely populated urban areas, with a 25% increase in their number reported in 2024 and a 14-fold increase noted in the past decade (12). Approximately 13,151 slums are distributed across approximately 734 major cities in all 27 Brazilian states (13). Although a recent study by our research group detected no anti-*Leptospira* spp. antibodies in homeless persons, leptospirosis and its associated factors in this vulnerable population remain to be fully established. Accordingly, this study aimed to assess the seroprevalence and factors associated with detection of pathogenic *Leptospira* spp. Antibodies among persons experiencing homelessness in urban areas in the two major cities of São Paulo and São José dos Pinhais in southeastern and southern Brazil, respectively.

## 2 Materials and methods

### 2.1 Study areas and sample collection

The present study was conducted in São Paulo city (23° 32′56′′ S; 46° 38′20′′ W), the largest city in Brazil and the southern hemisphere, with a population of 12.4 million people, and in São José dos Pinhais city (25° 31′51″S, 49° 11′45″W), a part of Curitiba, the eighth biggest metropolitan area of Brazil, ranked 85/5,565 (top 1.5%) in population, with 334,620 habitants (14, 15).

Sampling was performed in August 2020, after obtaining voluntary signed consent for participation during the coronavirus disease (COVID-19) pandemic. Homeless individuals have responded to an epidemiological questionnaire, providing personal information on their demographics, habits, addictions, medical and health status, and animal contact (Supplementary material 1). Blood samples were collected through venipuncture, performed by certified nurses, and placed into vacuum tubes and centrifuged, and the serum samples were stored at −20°C until processing.

### 2.2 Serological testing

Samples were tested using the microscopic agglutination test (MAT) to detect pathogenic *Leptospira* spp. antibodies at the Department of Veterinary Hygiene and Public Health, São Paulo State University, Brazil, in accordance with the Brazilian Ministry of Health protocols (16). A collection of 30 serovars stored at 28°C in Ellinghausen McCullough–Johnson–Harris medium was used as the testing panel. It included *Leptospira interrogans* serovars Australis, Autumnalis, Bataviae, Bratislava, Canicola, Copenhageni, Djasiman, Hardjo (type Prajitno), Hardjo (type CTG), Hebdomadis, Icterohaemorrhagiae, Nupezo-01, Pomona, Pyrogenes, Sentot, and Wolffi; *L. borgpetersenii* serovars Castellonis, Hardjo (type Bovis), Hardjo (type Minis), Javanica, Tarassovi, and Whitcombi; *L. kirschneri* serovars Butembo, Cynopteri and Grippotyphosa; *L. noguchii* serovar Panama; *L. santarosai* serovars Guaricura and Shermani; and the non-pathogenic *L. biflexa* serovars Andamana and Patoc.

As previously established, a 1:100 dilution was the cutoff point for determining exposure to *Leptospira* spp. If a sample was positive for more than one serovar, the one with the highest titer was considered as the causative infectious agent (16).

### 2.3 Statistical analysis

The variables were first chosen using unconditional logistic regression models for each variable, with a *p*-value ≤0.25. Subsequently, a conditional model was built using a forward strategy for variable inclusion, and Bayesian Information Criteria were used to assess the goodness of fit of the various models. For the variables included in the final model, odds ratios and their 95% confidence intervals (CIs) were calculated, and statistical significance was set at a *p*-value of 0.05, given the sample size. Additionally, biological plausibility was determined, and potential confounders were used to examine the interactions between the factors.

Three analyses were performed, one with a model considering all the positive MAT results and two others, with models considering the positive MAT results for serovars of pathogenic and non-pathogenic *Leptospira* spp. separately. All statistical analyses were conducted using the R (V.4.1.2) software (13).

### 2.4 Ethical considerations

This study was approved by the Ethics Committee of Human Health of the Federal University of Paraná, Brazilian Ministry of Health (CAAE:80099017.3.3004.0086; protocol number: 3.366.684). All research participants voluntarily signed written informed consent.

## 3 Results

In total, 21/243 (8.6%; 95% CI = 5.6–13.1%) persons experiencing homelessness were seropositive in the MAT. The results were analyzed and are presented in Tables 1, 2 by serovars and titers. In addition, the potential individual risk factors were assessed for all reacting serovars (Table 3). The final model included three variables (location of the person, use of condoms during sexual intercourse, and flea infestation) and one interaction term (interaction between the use of condoms during sexual intercourse and flea infestation). Significant associations (*p* < 0.05) were observed for all the three variables tested. Regarding the location of the person, persons in São Paulo presented a lower likelihood of exposure to leptospires than did those in São José dos Pinhais.

**Table 1:** Distribution of positive reactors to the different serovars of *Leptospira* spp. included in the microscopic agglutination test (MAT), from persons experiencing homelessness living in two cities of Brazil (São Paulo and São José dos Pinhais).

Type	Serovar	Total reactors (n)	% and 95 CI
Pathogenic		11	5.3 (3.0–9.2)
	*L. interrogans* serovar Bratislava	1	0.4 (0.1–2.6)
	*L. interrogans* serovar Canicola	4	1.6 (0.5–4.4)
	*L. interrogans* serovar Pyrogenes	2	0.8 (0.1–3.3)
	*L. kirschneri* serovar Grippotyphosa	4	1.6 (0.5–4.4)
Non-pathogenic		10	3.3 (1.5–6.6)
	*L. biflexa* serovar Andamana	8	1.3 (1.5–6.6)
	*L. biflexa* serovar Patoc	2	0.8 (0.1–3.3)

**Table 2:** Distribution of the titres of antibodies to the different serovars of *Leptospira* spp. included in the microscopic agglutination test (MAT), from homeless people living in two cities of Brazil (São Paulo and São José dos Pinhais).

Type	Serovar	Titre	(n)
		1/100	1/200
Pathogenic			
	*L. interrogans* serovar Bratislava	0	1
	*L. interrogans* serovar Canicola	3	1
	*L. interrogans* serovar Pyrogenes	2	0
	*L. kirschneri* serovar Grippotyphosa	4	0
Non-pathogenic			
	*L. biflexa* serovar Andamana	8	0
	*L. biflexa* serovar Patoc	2	0

**Table 3:** Conditional logistic regression model results showing the factors associated with infection by *Leptospira* spp. in persons experiencing homelessness from São Paulo city, Brazil.

Variable	Categories	Frequency in seropositive individuals	OR	95% CI	*p*-value
City	SJP	—	Ref.	—	—
	São Paulo	—	0.22	(0.07–0.64)	0.005
Use of condom during sexual intercourses	No	10/21 (47.6%)	Ref.	—	—
	Yes	9/21 (42.9%)	0.28	(0.09–0.90)	0.032
Flea infestation	No	16/21 (76.2%)	Ref.	—	—
	Yes	5/21 (23.8%)	0.09	(0.01–0.77)	0.028
Condom use (yes) and flea infestation		—	12.3	(0.97–157.0)	0.053

The final conditional logistic regression model for the factors associated with the serological status of reacting pathogenic *Leptospira* serovars was constructed (Table 4) to include two variables: the use of condoms during sexual intercourse and frequency of alcohol and drug consumption. The final conditional logistic regression model for the factors associated with the serological status for all reacting non-pathogenic *Leptospira* serovars included only one variable: location of the person (Table 5).

**Table 4:** Conditional logistic regression model results showing the factors associated with infection by pathogenic *Leptospira* spp. in persons experiencing homelessness from São Paulo city, Brazil.

Variable	Categories	OR	95% CI	*p*-value
Use of condoms during sexual intercourses	No	Ref.		
	Yes	0.26	(0.07–1.02)	0.054
Consume of Alcohol & drugs	No	Ref.		
	Yes	0.23	(0.03–1.95)	0.028

**Table 5:** Conditional logistic regression model results showing the factors associated with infection by non-pathogenic *Leptospira* spp. in persons experiencing homelessness from São Paulo city, Brazil.

Variable	Categories	OR	95% CI	*p*-value
City	SJP	Ref.		
	São Paulo	0.02	(0.003–0.20)	0.054

BIC = 60.70; SJP = São José dos Pinhais.

## 4 Discussion

The present study is the first to report a high exposure to leptospirosis in persons experiencing homelessness. Homelessness has been considered one of the most vulnerable situations worldwide, alongside deprivation of liberty (incarceration) and displacement from home country (refugees) (17). Structural inequality and exposure to diseases, such as leptospirosis, may be aggravated by individual issues, including drug addiction, mental disorders, and improper healthcare (18). In addition, the COVID-19 pandemic significantly worsened the situation of homeless people globally, particularly in Brazil where it caused approximately 700,000 deaths (19). Such overlapping exposure to different infectious diseases caused by various infectious agents, including waterborne (leptospirosis) and airborne (COVID-19) pathogens, may come as no surprise as persons experiencing homelessness were among those affected the most worldwide during the pandemic (20). Furthermore, the lack of epidemiological data on persons experiencing homelessness and the potential delay in healthcare access may result in underdiagnosis and uncontrolled disease spread, particularly of sexually transmitted infections (10, 21). Moreover, discrimination and prejudice, coupled with structurally inadequate reporting and database systems, may have obstructed healthcare services, with disastrous results (22).

This study is the first to indicate that persons experiencing homelessness in both sampled cities are highly exposed to leptospires, with a frequency of 10.3% in São Paulo and 17.5% in São José dos Pinhais. Interestingly, a recent study by our research group found no seropositive samples out of 200 samples collected from persons experiencing homelessness in São Paulo, Curitiba, and Foz do Iguaçu (16); this was explained at the time by nomadic homeless behavior, moving to higher areas during flooding, and avoiding water potentially contaminated by rat urine, which may have prevented exposure to bacterial infection. As this study was conducted in the same areas but during the COVID-19 pandemic, looking for food and personal supplies (clothes) may have imposed behavioral changes, increasing the exposure to *Leptospira* spp. in persons experiencing homelessness in Brazil and worldwide. Thus, the pandemic may explain the rapid fluctuation in *Leptospira* spp. seroprevalence within a 3-year period using the same testing method and laboratory and in similar homeless populations. In addition, our previous report found that 6.7% of seropositive (non-vaccinated) dogs were owned by persons experiencing homelessness (16); therefore, this *Leptospira* infection source should be considered. Unfortunately, sampling of dogs was not allowed by health authorities for this second sampling, mostly because of the necessity of restraining the dogs and assistance by homeless owners, under the total absence of available human COVID-19 vaccinations at the time (16).

The results herein found for persons experiencing homelessness were not surprising, as Brazil has been historically recognized as an endemic country for leptospirosis, with 3,810 cases reported on average per year. The majority of affected individuals live in densely populated urban areas (23), where persons experiencing homelessness mostly live, under poor conditions (16). Although rural clusters of leptospirosis in Brazil have been associated with large-scale agricultural practices and animal husbandry (23), countryside settlements of landless people should be further investigated for leptospirosis, as already carried out by our research group for toxoplasmosis (24), ehrlichiosis (25), hemotropic mycoplasmas (26), and borreliosis (27). In the present study, there were seropositive samples of non-pathogenic leptospires, with a higher prevalence of the Andamana serovar. However, the pathogenic potential of this serovar is still debated, as evidenced by a fatal case reported in a sewage worker from the city of São Paulo, in which *Leptospira Andamana* was identified in the cerebrospinal fluid (3).

As mentioned above, sampling was conducted in August 2020, with Brazil being the worldwide epicenter of the COVID-19 pandemic, which may have severely impacted infectious disease exposure due to the simultaneous lack of proper food, shelter, and healthcare assistance at the time (20). Unfortunately, owing to a lack of resources and safety (no vaccines) during the pandemic, only homeless people living in São Paulo city (and no dogs) were sampled and serologically tested. This carefulness was fully justified, as almost 56% of persons experiencing homelessness were found to be seropositive for COVID-19, the highest prevalence ever registered worldwide (20). The impact of the COVID-19 pandemic on leptospirosis was assessed, with 9.1 and 6.7% seropositivity reported without and with the use of face masks, respectively. In addition, individuals tested for COVID-19 exhibited a 9.5% positive reaction rate for leptospirosis, compared with 8.6% among those not tested. Thus, the present study may also serve as a warning for the potential for co-infection and an increase in leptospirosis exposure, not only following flooding but also during public health catastrophes, which could lead to an increase in the vulnerability of such populations.

Leptospirosis is closely related to social determinants of health and is mostly associated with flooding, precarious living conditions, and rodent infestation (23). Persons experiencing homelessness and residents of urban slums worldwide face higher exposure to infectious diseases due to unfavorable socioeconomic conditions, lack of adequate sanitation, clean water, and suitable housing, which are associated with low education levels and informal work or occupations (28). Effective public health strategies for combating leptospirosis should consider the complexity of socioeconomic and environmental factors, accompanied by routine detection, mapping, control, and preventive measures (29). In such a scenario, a One Health approach for leptospirosis is essential for a better understanding of disease impact and control on human, animal, and environmental health (30), particularly zoonotic diseases, such as leptospirosis, which have been strongly linked to environments infested with rats (30). Such a multidisciplinary approach is crucial for identifying the associated factors as well as the role of outdoor activities, contact with contaminated soil, and proximity to rodent habitats for infection (31).

The distribution and diversity of *Leptospira* spp. serovars among persons experiencing homelessness indicated a prevalence of 5.3 and 3.3% for *L. biflexa* serovars Bratislava and Andamana, respectively, followed by 0.4 to 1.6% for *L. interrogans* serovars Canicola and Pyrogenes, and low detection of *L. kirschneri* serovar Grippotyphosa, and *L. meyeri* serovar Semaranga. These serovars have also been reported in rats from Brazil (32). Such a wide range of *Leptospira* spp. diversity has been attributed to host-species interactions and the influence of environmental factors, as observed in both stray dogs and patients with leptospirosis in the nearby northern Paraná state (31). Persons experiencing homelessness may be interpreted as environmental sentinels for the presence of pathogenic *Leptospira* spp. serovars, thus providing practical on-field mapping for more effective surveillance, control, and prevention measures for public and animal (domestic, livestock, and native species) health.

The final model revealed three statistical significant variables: location of the person, use of condoms during sexual intercourse, and flea infestation. Firstly, persons experiencing homelessness living in São Paulo presented a significantly lower *Leptospira* spp. seroprevalence than did those living in São José dos Pinhais (*p* = 0.005), which may be due to varying individual characteristics across different shelters and locations, as previously observed in Denmark (33). In addition, the Homeless Reference Center in São José dos Pinhais is situated in a less central location, adjacent to wetlands and wooded areas, with exposure to sewage, flooding, and domestic and wildlife hosts, overlapping with highly urban and green areas. In contrast, the shelter (day-only) in São Paulo city, named the Community Center of São Martinho de Lima, is located in a highly urbanized, low-income area, exposed to seasonal flooding and rats, but fully pavemented, within a highly commercial neighborhood (34). Thus, as expected, leptospirosis exposure in persons experiencing homelessness may vary under different socioeconomic conditions and among major urban cities, demanding continuous assessment to establish the local prevalence and pinpoint the exposure dynamics over time.

Another significant variable was the use of condoms during sexual intercourse, which was considered a protective factor against exposure to leptospires (*p* = 0.032). Although human-to-human transmission of *Leptospira* spp. is rare, infection through sexual intercourse and intimate contact may occur under unsanitary conditions (35, 36). In addition, condom use may indicate higher self-care and hygiene, which may reduce exposure to potential sources, such as rat urine and flooding. Conversely, flea infestation may indicate a more nomadic lifestyle, which may lower the exposure risk. Nevertheless, the present study found unusual factors associated with exposure to *Leptospira* spp., including a high self-reported proportion of persons experiencing homelessness, seeing rats, and experiencing rat bites. The presence of ectoparasites, such as lice and fleas, has also served as a warning for the complex dynamics between homelessness, zoonotic agents, and environmental factors. Although our research group has already found an association between lice infestation and 72.5% seropositivity for *Bartonella* spp., along with 36.7% seropositivity for the Rickettsia genus of the typhus group (37), the other findings presented here should be further investigated. The only study available for comparison till date, from a low-income neighborhood in Vancouver, Canada, showed 3.0% exposure among inhabitants to *Bartonella tribocorum* associated with local rats but no seropositivity to *L. interrogans* (38).

Surprisingly, persons experiencing homelessness with frequent alcohol and drug consumption herein were significantly less likely to be seropositive (*p* = 0.028) for pathogenic serovars of *Leptospira* spp. As previous studies have shown association of alcohol consumption with *Leptospira* infection in Thailand (39), Kenya (40), Seychelles (41) and Mexico (42), possibility of confounding factors or biased measurement should be considered in the present study. Nonetheless, further studies should be conducted to fully establish the associated risk factors of leptospirosis in Brazil.

Persons experiencing homelessness in São Paulo city had a lower likelihood of exposure to non-pathogenic *Leptospira* spp. than did those in São José dos Pinhais. Experimental studies in mice have suggested that saprophytic species of *Leptospira* may triggers innate immune responses in the host during early infection, leading to better outcomes after infection by pathogenic species (43). As both pathogenic and non-pathogenic *Leptospira* can exhibit similar survival capabilities and range of environments (44), further longitudinal studies may be necessary to confirm directionality and causality. In addition, other variables, including demographic variables, COVID-19-related factors, and medical variables were also analyzed, with a higher prevalence of leptospirosis observed in São José dos Pinhais (15.5%) than in São Paulo (6.9%). Although individuals of indigenous ethnicity remained negative, the positive reaction rates among Black, Brown, and White individuals were 5.4, 7.8, and 12.5%, respectively, confirming the diverse ethnic outcomes associated with leptospirosis. Previous surveys in Brazil have found a higher prevalence among White (45) and mixed-race individuals (46), with similar prevalence variability based on predominant regional ethnicities found in other countries (47, 48). In this study, 10.7% of the affected individuals were female and 8.6% were male, with no higher male prevalence, as previously observed (49, 50). In addition, age was similar between the groups, with 10.3% of the affected individuals aged under 30 years and 8.3% aged over 60 years. Sex and age are crucial factors to be considered when formulating effective and integrated control and prevention strategies, which may improve our understanding of zoonotic risks and mitigate exposure to infection in both human and animal populations (51).

Although reportedly an important protective factor, educational level was not significantly associated with *Leptospira* seropositivity, with 8.3, 9.1, and 8.4% of the affected individuals having completed higher education, high school, and eighth grade, respectively. The challenges posed by leptospirosis may require a multifaceted approach, raising public awareness of the associated factors and implementing preventive measures (28, 52). Continuing health education may play a pivotal role in enhancing the population’s knowledge of leptospirosis and improving its diagnosis, control, monitoring, and prevention (29).

The “Street Clinics” program, launched in 2011, aims to bridge healthcare disparities in homelessness in Brazil (22). Despite being successfully established, the program still faces challenges, including inadequate healthcare training and constant variations in epidemiological data (53). Moreover, the program has systematically applied rapid tests only for sexually transmitted diseases (acquired immunodeficiency syndrome [AIDS], hepatitis, and syphilis), with no regular screening for zoonotic diseases despite high drug use and sharing, unprotected sex, and low self-hygiene. Additionally, program participants exhibited lower seropositivity (5.7%) than non-participants did (10.2%), further illustrating the challenges faced and potential areas for improvement for the program. Pet owners demonstrated a lower positive reaction rate (5.0%) than did those without pets (10.5%). A similar pattern was observed in individuals with animal hoarding disorder, where the prevalence of leptospirosis among dogs was notably higher than that among owners (54). Assessment of medical variables revealed seropositive reactions for AIDS, syphilis, hepatitis, tuberculosis, diabetes, and cardiovascular diseases. In such a multi-exposure scenario, human and dog leptospirosis have been neglected in comparison to other diseases, despite their higher mortality rates (55). Finally, the high leptospirosis seropositivity in persons experiencing homelessness living in two major Brazilian cities has highlighted the need for intervention and awareness in a holistic approach to human, animal, and environmental health (One Health); collaborative endeavors are imperative to address such complex issues, as already proposed (56).

Although the herein sampling of 243 individuals in two major cities may be considered relatively low, limiting the statistical analysis and extrapolation of findings, homeless persons have been reportedly indicated worldwide as difficult population to assess, mostly due to mental illness, alcohol and drug abuse, leading to unsocial and erratic behavior, recluseness, and refusal to participate. Sampling was also limited to two urban locations within few days due to São Paulo as worldwide epicenter of the COVID-19 pandemics, without available vaccination at the time. Thus, broader geographic and seasonal assessment should be considered in further studies to ensure representativeness and extra polatory outcomes. As another limitation, the voluntary nature of participation may have introduced self-selection biased outcome, potentially excluding the most marginalized (alcohol and drug abuse) or medically vulnerable (mental illness) individuals. Also, the cross-sectional nature of the study here has limited causal inferences regarding identified risk factors (e.g., condom use, flea infestation), as longitudinal studies would be needed to confirm directionality and causality. Some apparently contradictory findings have been presented, for example a previous study with no anti-*Leptospira* antibodies found in homeless persons of three major Brazilian cities, whereas significant seropositivity was observed herein with samples collected during COVID-19 pandemics. Although pandemics may indicate less chances of getting exposure because of restricted movement and other protective measures which were more or less prevalent, the same individuals have shown the highest worldwide seroprevalence of COVID-19 with 111/203 (54.7%) seropositive persons, with no available vaccine at the time (therefore no ELISA crossreaction) (20). Thus, homeless persons have not followed pandemics recommendations of self-distance, isolation and use of preventive discardable face masks. Likewise, the change in behavior of homeless persons may have increased their exposure to rats and *Leptospira* infection, likely due to gathering on sidewalk tents around the daytime shelters, which provided three meals per day through pandemics, maybe the only source of available food at the time. Finally, antibodies observed herein may be indicative of exposure a long time ago and not correlate with the time of sampling during COVID-19.

This study has shown that persons experiencing homelessness living in São Paulo and São José dos Pinhais, Brazil were highly exposed to leptospirosis, which may indicate nationwide exposure, demanding urgent surveillance, control, and prevention. Location, condom use, and flea infestation were identified as significant risk factors for leptospirosis exposure. The presence of multiple *Leptospira* serovars may have indicated bacterial diversity, particularly in urban settings. As seropositivity herein has involved both non-pathogenic and pathogenic species, the pathogenic potential should be carefully interpreted to avoid overinterpretation. Furthermore, multidisciplinary efforts and integrated policies may be crucial for mitigating leptospirosis and other infectious diseases in persons experiencing homelessness, as social neglection may impact their fundamental rights to health, shelter and food access. Finally, although public policies have been demanded by society and non-governmental organizations, the homeless situation has been multifactorial and far from a consensus solution. In such a scenario, priest Julio Lancellotti, who has been assisting homeless persons in São Paulo for the past 40 years and was personally involved in the homeless engagement herein, has blamed city approach to homelessness as “invisibles on street,” claiming their rights to “minimum life conditions, dignity and subsistence (57).

## Data Availability

The original contributions presented in the study are included in the article/Supplementary material, further inquiries can be directed to the corresponding author.
