# Supplementary material for: Factors associated with the presence of anti-Leptospira spp. antibodies in persons experiencing homelessness in Brazil
Source: Front Public Health. 2025 Jul 11;13:1596684. doi: 10.3389/fpubh.2025.1596684 (PMC12289583; doi:10.3389/fpubh.2025.1596684)
Supplement: Supplementary file 1 [file Supplementary_file_1.docx]

Supplementary Material

# Supplementary Material 1

Full name:

City of Origin:

Date of Birth (day/month/year):

Gender: ( ) Male ( ) Female ( ) Other:

Ethnic Background: ( ) White ( ) Black () Mixed Race ( ) Indigenous

Education Level: ( ) No Literacy ( ) Incomplete Primary School ( ) Complete Primary School ( ) Incomplete Elementary School ( ) Complete Elementary School ( ) Incomplete High School ( ) Complete High School ( ) Incomplete Higher Education ( ) Complete Higher Education ( ) Postgraduate Studies

Assisted by the "Consultório na Rua" (Street Clinic) program? ( ) Yes ( ) No

Assisted by CAPS (Psychosocial Care Center)? () Yes, which one? _____ ( ) No

Do you use chemical substances? ( ) Yes ( ) No

Alcohol consumption ( ) Yes ( ) No

Tobacco use ( ) Yes ( ) No

Marijuana use ( ) Yes ( ) No

Cocaine use ( ) Yes ( ) No

Crack use ( ) Yes ( ) No

Injectable drugs use ( ) Yes ( ) No

How long have you been on the streets (years)?

Do you stay in this city permanently, or do you move around? ( ) Permanent ( ) Moves Around

Source of drinking water: Public water supply ( ) Yes ( ) No; Well ( ) Yes ( ) No; Rainwater ( ) Yes ( ) No; Other:

Do you eat raw meat? ( ) Yes ( ) No

Reason for going to the streets? Unemployment ( ) Yes ( ) No; Alcohol/Drugs ( ) Yes ( ) No; Family Conflicts ( ) Yes ( ) No; Other:

If female, are you pregnant? ( ) Yes ( ) No

Do you use condoms during sexual relations? ( ) Yes ( ) No ( ) Sometimes

Do you use a face mask? ( ) Yes ( ) No

Have you had contact with someone suspected/positive for COVID-19? ( ) Yes. When? _______ ( ) No

Have you presented any of the following clinical signs in recent months? ( ) fever; ( ) sore throat; ( ) difficulty breathing; ( ) diarrhea; ( ) fatigue; ( ) chest pain/pressure; ( ) dry cough; ( ) loss of smell/taste; ( ) headache; ( ) body aches/discomfort; ( ) other _______

Were you examined? ( ) Yes. Observations: _______ ( ) No.

Were you tested for COVID-19 (SARS-CoV-2)? ( ) Yes. When? _______ ( ) No

If yes, which test? () Rapid test (blood). Result: ___ ( ) PCR (swab). Result: ___

Do you have companion animals? ( ) Yes ( ) dog(s), how many? _____ ( ) cat(s), how many? ____ ( ) other(s); ( ) No

Do you have contact with soil or sand? ( ) Yes ( ) No

Do you have a nail-biting habit? ( ) Yes ( ) No

HIV? Declaration: ( ) Positive ( ) Negative

Syphilis? Declaration: ( ) Positive ( ) Negative

Hepatitis? Declaration: ( ) Positive ( ) Negative

Cardiovascular Disease ( ) Yes ( ) No

History of Tuberculosis: ( ) Yes ( ) No

History of Diabetes: ( ) Yes ( ) No

Other illness? ( ) Yes, which one? ____ ( ) No

Do you have access to soap and water to wash your hands daily? ( ) Yes ( ) No

Frequency of bathing: ( ) Daily ( ) 2 times a week ( ) 1 time a week ( ) 1 time a month ( ) Not informed;

Frequency of changing clothes: ( ) 2-4 times a month; ( ) 2-3 times a month; ( ) 1 time a month

Bitten by "muquirana" (body lice)? ( ) Yes ( ) No

Have you ever been bitten by fleas? ( ) Yes ( ) No

Do you see rats in your resting place? ( ) Yes ( ) No

Have you ever been bitten by rats? ( ) Yes ( ) No
